# Fragments of Medical Science and Art

**Published:** 1846-04

**Authors:** 


### ARTICLE IV.

*Fragments of Medical Science and Art.* An Address delivered before the Boylston Medical Society of Harvard University. By Henry Jacob Bigelow, M.D. Published by the Society. pp. 54. (From the Author.)

To know one's ignorance is the first step to improvement; an axiom as fully applicable to a science as to an individual. Were it possible that the true condition of Medical Science could be known to all its votaries, not only would it advance with more rapid strides towards perfection, but its advance would then be real, while alas! it is now, but too often, apparent. When we see the once apparently established theories of age after age, crumbling into atoms and leaving but a mass of rubbish to impede the progress, and to be cleared away by future or present laborers, we are naturally lead to enquire into the causes of this want of durability. The subject matter of the above Address is, in general terms, an exposition of these causes, and of the true mode of conducting pathological investigations. The Address commences with a review of the Baconian system of philosophy, and its applicability to medical science. In speaking of the sources of false systems the author remarks:

"It does not require patient investigation and laborious thought to frame a false system of philosophy, to deduce an ingenious theory from a few facts; but truth unveils herself to constancy alone, to persevering love, of which few are capable. Nor is it the cause of error to which man, once enlisted, clings with such tenacity; he clings to his own cause; his own philosophy; with the determination of a chief or the zeal of an adherent; pride animates and prejudice arms him; it is the cause of human passion. It is not then wonderful that there are so few minds able to divest themselves of human weakness, to acknowledge error and abandon cherished fallacy, to love truth for its own sake; and to find in its discovery a source of elevated happiness."

A few remarks upon false generalization, showing the fertile soil producing the many quackeries of the age, we may also quote.

"The inductive method also teaches, that if truth lies in facts, we have only to collect facts to discover it. But Bacon knew how ready the mind is to pursue some false light into the morass of error; 'to start off to generalities, that it may avoid labor.' To insure accuracy, therefore, facts are to be written down and subsequently counted. In Bacon's own words, 'we must form tables and coördinations of instances, upon such a plan and in such order that the understanding may be enabled to act upon them.'* All this is now obvious and undoubted. False theory is not the error of our day, nor of our scientific community. But while in medicine, the value of facts is recognized, possibly overrated, their application is a fertile source of error.

"Open any medical journal of the last week, or of last year, and you shall find an account of some new, successful remedy; and yet another year shall obliterate this record, and substitute new methods equally infallible. There is error either in the facts or in the induction. Here is Bacon's quaint exposition of this fallacy. 'It was well answered by him who was shown in a temple the votive tablets, suspended by such as had escaped the peril of shipwreck, and was pressed as to whether he would then recognize the power of the gods, by an inquiry, 'But where are the portraits of those who have perished in spite of their vows?''† We listen to partial or imperfect evidence. Impressed with an idea, we accept only the facts whose tendency confirms our notion. In the words of Bacon,‡ 'It is the peculiar and perpetual error of the human understanding to be more moved and excited by affirmatives than negatives, whereas it ought duly and regularly to be impartial; nay, in establishing a true axiom' (or law) 'the negative instance is the most powerful.' These natural tendencies of the mind, then, show the necessity of insuring accuracy by writing; 'the understanding being as incapable of acting on such materials of itself, with the aid of memory

* Nov. Org. B. 2, Aph. 10. † Id. B. 1, Aph. 46. ‡ Id. B. 1, Aph. 45.

alone, as any person would be of retaining and achieving by memory the computation of an almanack.' 'Yet meditation,' says Bacon, and with almost equal truth might it have come from Louis, 'has hitherto done more for discovery than writing, and no experiments have been committed to paper.'*

"In its most general sense, induction is not only applicable, it is essential to the discovery of all law in nature. The student admits this, and yet at some time or other most of us have felt a certain undefined doubt whether the inductive method was adapted to the wants of medical science."

After premising a general view of the inductive system of philosophy, the author proceeds to discuss certain questions of interest in connection with medical science, "and first, How much of medical science is drawn from fact by process of induction, and how far are we able to anticipate this process, and to fortel certain facts by means of others?" In regard to the three forces governing the laws of nature, viz: "mechanical, chemical, and, in a more limited degree, polar force," the inductive system is indisputably applicable, and the only true mode of scientific investigation, and, of course, where they are found to have a bearing upon medical science, that system must be followed in the investigation of medical laws. But in addition to these forces in medical science "there are vital forces at work which counteract the most obvious tendencies of the material particles of the human fabric. What prevents the arteries from being dyed red, or the body from falling to the ground, or in short the whole mass from decomposing? It is vital force, whose power to hinder the most obvious effects of other force must, till we comprehend its nature, render all prediction in the vital science fallible."

We continue to quote the author, as his own language is as much condensed as the nature of the subject will permit and we are loath to leave out one word of it.

"There are but few sciences in which we have detected the machinery which is the immediate cause of their phenomena; and although, in the words of Bacon, 'It is rightly laid down that true knowledge is that which is deduced from causes,'† many sciences like ours are made up merely of aggregated facts, without reference to the manner of their production. Laws of phenomena are the sum and expression of knowledge already acquired; they open no new region of knowledge. Why do we know that lymph is and will be common on the tonsils and in the air tubes? Because we have observed it. But what does this tell us of the intestinal canal, or other mucous surfaces, where it is comparatively rare? It tells us nothing; we know only what we have known. Our knowledge is limited by the fact; and with blind reliance we trust in

* Nov. Org. B. 1, Aph. 45. † Nov. Org. B 2, Aph. 2.

nature's constancy, and in the accurate adjustment of her vast machinery. Far different is the science which studies the detail of the great machine, which understands its complex movements and detects its variations, which wrests from resisting nature her great secret, and fortels, ages beforehand, the workings of her universal laws.

"Yet have these laws of phenomena a great and undisputed value. The facts, in the words of Kepler, 'are collected into one fagot,' though no scrutiny has enabled us to penetrate their substance. Laws of phenomena detect and assert the similarity of facts, they point out their common element, and draw from each a thread by which they are suspended from a common centre. In time these threads are twisted into cords, which in their turn are woven into some great fabric. They distinctly state a proposition which is to be subsequently proved or disproved, and supply a series of equations from which the value of the common $x$ is finally deduced. In the instance, for example, of polarity, a force now in process of discovery, we thus trace the connexion of the ray of the setting sun as it was reflected from the windows of the Luxembourg with the phenomena of crystallization, chemical affinity, magnetism and electricity. Wild and straggling facts of unknown growth are here laid side by side. They are brought into the domain of science, and the wedge is at last entered which lays them open and reveals their hidden entity.

"The chief employment of the medical philosopher is to establish these laws of phenomena; to ascertain the frequency with which certain facts occur with other facts; to point out the element most constant to a wide range of phenomena; the very ends which the inductive method of Bacon, and the numerical method of Louis, its medical application, teach us to attain it with certainty. Medical diagnosis and medical prognosis obviously grow out of rigid induction.

"But let us note an important distinction in the present application of this return to diagnosis and to therapeutics. If we err in our principles of diagnosis, it is of comparatively little consequence. If we choose to consider sore-throat a necessary effect of the cause which produces the other symptoms of scarlet fever, or the eruptions essential to the exanthemata, when they are really only incidental to them, science alone suffers. The inductive method offers us the surest results we can obtain, even though these are uncertain. But if we err in therapeutics, it is not science alone, it is the patient that suffers. Suppose it is conclusively shown, that when a hundred patients are bled in a certain disease, fifty-five always recover; and that when these hundred patients are not bled, only fifty get well; and suppose we bleed our hundred patients; we shall be very likely to kill somebody who, otherwise treated, would have proved a useful member of society.

Science gains her five per cent, but humanity loses a man or a woman or a child. Such treatment may do for armies, where one man is as good as another; but does not answer for individuals, by nature prone to over-estimate their personal consideration.

"The fact is, the diseases we compare, and whose similarity we affirm, are very dissimilar. Every case of disease varies in its character from every other case of the same disease. It varies in its intensity, its complications, and its tendencies. It varies with the constitution of the patient, and with external influences. It varies in the rapidity of its march, and in the duration of each symptom. The same treatment, therefore, affects single cases very differently. But it is a singular principle in nature, that differences tend to neutralize each other in the long run. That while individuals deviate, like the asteroids in their revolutions, equally above and below a fixed line, the tendency of the mass is to an average always constant. This principle equalizes the number of births and deaths; of male and female children; it makes the annual proportion of dead letters to other letters always the same in the English post-office; and in exactly regulating the laws of mortality, it guarantees success to life insurance companies founded on proper computations. But it does not tell us the sex of the child, nor the length of any man's life, nor the chance any given letter has of being called for. Men vary above or below a certain healthy standard. If in some disease this standard is too high, it must be lowered, but in this process some one whose scale of mortality is already low will be submerged, yet we cannot tell who this individual will be, until we know what constitutes vital force. No rule in therapeutics applies indiscriminately to any other than a theoretically fixed standard of disease; if we can arrive at such an ideal standard by only averaging the mass, the rule is applicable only to this mass; and so long as the practice of medicine is for the benefit of individuals, such a principle will be of limited utility to the physician. To give fair play to this equalizing tendency, induction should be based upon a broad range of facts. Narrow induction is the bane of medical science, and the physician who infers that nature will gain her equilibrium in three or five successive facts, and argues from them, might with equal wisdom, in a game of chance, bet upon *rouge*, because it has won but twice, while *noir* has gained three times.

"But let no one argue for these reasons the inadequacy of the inductive method. It is ignorance alone that hinders us from availing ourselves of it. If medicine were an exact science it would be not only applicable but necessary to therapeutics, and as science advances, and we approach nearer and nearer this stage of medical knowledge, induction becomes more and more indispensable. And even now the self-adjusting tendency of nature furnishes us truth in its relation to the mass. The inductive method is of undisputed value in determining, for example, whether a supposed remedy has or has not a value. More than this it tells us very nearly what this value is. If fifty patients in a hundred have recovered from a certain malady under the use of carbonate of iron, and fifty in a hundred have got well without it, I decide this remedy to be nearly inert in this diease. If the proportion of the recoveries under the remedy much exceed this statement, I infer that the medicine possesses some degree of efficacy; if the number of recoveries is much less, I am equally sure it is injurious. And I am right in all these suppositions. But there is another quality of the inductive method, and I am not aware that it has been observed, which requires that the results derived from it, at least in therapeutics, should be of unequivocal numerical force. The inductive method speaks of coincidence and not of cause. The lesion of Peyer's glands is coincident with febrile symptoms in typhoid fever, and so also is sore throat in scarlet fever; but we have only probable evidence whether either of these conditions is a cause or an effect. Now this relation is essential in therapeutics, we require that a remedy should seem to be a cause of convalescence, and not merely a coincidence. If with Hildanus I anoint the axe that made the wound, and fifty-five instead of fifty per cent. of wounded knights recover, does the additional five per cent make me believe in the efficacy of the "*unguentum?*" Can I believe in the therapeutic agency of dead man's touch, or

"Slips of yew silvered in the moon's eclipse,
Nose of Turk and Tartars's lips."

Reason is against it, in spite of any possible and obvious cajolery of the inductive method. In the connexion of remedy and disease, we must see a probable, or at least not impossible relation of cause and effect; and whence shall we derive this evidence? It must be supplied by the mind of the observer, and based either upon previous general or medical experience, or else upon the evidence of the immediate experiment, which must, for this end, be of overwhelming force.

To be applicable even to the mass, the evidence should be of weight, and it has been shown that unless the evidence be conclusive, and hold good of all the individuals upon whom the experiment is tried, we have no right to apply to individuals that which is true only of the mass. We can follow no indiscriminate rule in therapeutics. We can only pause and weigh the indications, and decide each case upon its own requirements. It was a common saying of the late Dr. Baillie, 'Learn your profession well, and practice it on rule of common sense;'* and it cannot be denied, that in the therapeutics

* Observations in Medicine, Marshall Hall, Chap. 2.

of the present day, the physician must rely to a great extent on the resources of his judgment. And what is judgment? It is the faculty which God has given man to supply the place of certain knowledge. Because invading pneumonia must be bled, will you bleed the wretch already lingering on the borders of the grave? Judgment is the act of common reason; it is a logical decision based upon the evidence of facts; and is this faculty impossible to you? Shall not your hundred cases of fever, analysed, combined, weighed, sifted in the wards of a hospital, be to you at least as the 'experience' of a hundred cases, scattered among the engrossing occupations of twenty years? Do not struggle with the crushing incubus, distracting you with its monotone, that judgment and experience come only with age. Is medical science the only one beyond the reach of active intellect? Is the mind to be depleted, and the vital force exhausted down to the comprehension of the vital science? But I am encroaching upon ground I intend to occupy at the latter part of these remarks."

Pages 19 to 32 discuss the value of hypotheses, and show in a clear and lively style, the proper mode of their construction and their abuse. The danger of abuse our author correctly refers to the devotion of the investigator to his favorite theory, —the feeling of parentage—as may be seen in the following:

"But facts are not to be perverted. Their use is to test, to prove or disprove, not to confirm theory. The error of former science, the error of recent medicine, was not that hypotheses were made, but that they were constructed to be proved; that truth was warped and misapplied; that facts were prostituted in the cause of ignorance and prejudice. Philosophers made a show of listening to the evidence of nature, while they aimed to prove a verdict, which was signed and sealed before the trial. Such, in our science, were the 'inflammation' of Broussais, the febrile theory of Cullen, the stimulating system of Rasori, and many other theories, which first stated a proposition, and then collected facts to support and confirm, not to test it."

The success of great discoverers in unraveling the intricate mazes of scientific phenomena, is referred to their inveterate love of truth, which lead them to reject, at once, one hypothesis, the moment it is contradicted by fact.

The Address goes on to show the character of mind most successful in building up hypotheses, and conducting them to correct conclusions. We have, upon this part of the subject, but space for a few short remarks of our author. He says, "I have tried to show that it is not the ability to sum up the common elements of facts arranged in tables, nor to verify hypothesis, that adapts a mind for observation. I will add that is not the mere power to make a theory. The theory must be of a certain quality; a probable if not a successful one. * * * * "But while facility in forming probable theory is distinctive of scientific genius in the mind of the philosopher, the love of truth is steadfast and predominant. No ingenuity of reasoning, no parental affection for theory, no human passion, turns it from the contemplation of reality. It never shrinks from the untold labor of investigation; it is hard upon the tracks of truth, and staunch in its pursuit."

The value of *facts* and the uses to be made of them, forms the next subject of our author, and, while he recognizes their value as the means of establishing a starting point for the inductive philosopher, he condemns the tendency of the age to amass facts without connecting them with causes, or their relations to medical laws. In this regard our author gives the French school the credit of superiority. His advice in this connection, if followed by contributors to medical periodicals, would, we think, give to their communications a value which they do not, in general, possess.

"But facts are not the end of science, and you are not to amass them blindly. Have a purpose in your investigations; let it be either of self education, of verifying for yourselves what is already established, of forming an hypothesis, or of testing one. Do not think that impartiality in observation requires that a fact should stand alone; should offer no indication of the existence of any other fact. Rather endeavor to find some true relation between a symptom and its immediate cause, between a sign and any alterations of structure or of function, between one disease or lesion and another. Here, if I may say it, there seems to me to be a difference in the tendencies of medical knowledge in this place and in the French school.* The connection of medical phenomena is there, if possible, determined. Every fact is labelled with its scientific value. There, a true relation is, if possible, indicated; here, if possible, it is evaded. The details of a medical case may be narrated or recorded promiscuously and without order, or methodically, to illustrate its bearings upon certain points. Either history will embrace all the facts; but while one supplies only the crude material, the other shapes the block and designates its place in the future edifice, and leaves to future science only the labor of adjusting it. The ill digested observation is in fact comparatively useless. Medical discovery has generally been the work of individuals, who had their hypotheses and have analyzed facts in some especial relations, and not of societies, who have collected them indiscriminately. But if any discovery is ever to be thus made, if observations thus accumulated by different individuals are ever to be collected, and studied together, especially when we remember the diffi-

* See Appendix C.

culty and almost impossibility of analyzing a wilderness of medical facts matted and felted together into a dense and tangled mass, let us be ready to advance the labor in our humble spheres, at least in showing what points our observations are intended to illustrate, and in adopting a methodical and condensed arrangement which shall facilitate their future comparison."

The Address, in defining the position of Medical with other departments of Science, acknowledges its imperfections, and ascribes it to the difficulty of recognising the modes of action of the *force of life;* that while the "astronomer's facts repeat themselves after a determinate interval; the chemist occasions his at will, the pathologist can only wait for promiscuous phenomena, whose occurrence is amenable to no known influences."

The usefulness of our profession is set forth as follows:

"Have I seemed to limit too narrowly the range of our professional usefulness? Will it be said, that I am treacherous to the cause of medical science, in confirming, by the evidence of an adept, what was already suspected by the uninitiated? The dignity of our science will not be diminished by a recognition of its real abilities. It is its actual condition, and not the avowal of it, that can alone add or detract from its real character. If our science has advanced little, in comparison with some others, it is because it is built upon them, and because its problems are combinations of the most difficult and unexplored of their laws. But if it is among the least advanced, it is among the most important. It wields many of the physical conditions of human existence. It has averted physical calamities, which would by this time have anticipated whole ages of aggregated human life. Were it for its single power of controlling sensation, our profession would be necessary to the civilized world. But we can do far more than this. The physician can foretel the duration of most acute diseases; and apart from the accidents of life, he promises health. He alone can judge, from a wide combination of symptoms, of the actual condition of the patient. He condemns without appeal. He can sometimes arrest disease, and can often modify symptoms, or mitigate their severity. In its true form, our science is already indispensable to man."

We have already extended our review of this admirable address much further than we had intended, but such has been our appreciation of its excellence, our admiration of the sterling truths which it contains, that our labor has not been in selecting passages of value and interest, but to determine what we should be most willing that our readers should lose, and extended as have already been our extracts, we cannot close without quoting some selected paragraphs of the portion of the Address, devoted to advice, as to the conduct of medical practitioners, their duties to the profession, their patients and themselves.

"True boldness is that which a sense of competency maintains. Weigh your ability, and decide whether you are competent to a responsible position. Do not confound with the question of ability, that feeling of repugnance, which every young man has in undertaking an untried duty. With a well grounded confidence, justice to yourselves demands the sacrifice of vague doubts and nervous hesitation. Endeavor to know in medicine what is to be known; and while you save yourselves much of the embarrassment which arises from a want of confidence, you will spare yourselves the necessity of dissimulating ignorance. There was a time, and that not far distant, when the grave and professorial air was a necessary part of our professional attainment; when an expression of profound sagacity hung as a veil before the mysterious recesses of the mind; and an air of deprecating merit seemed to restrain and to dam up unknown depths of wisdom. A good physician generated an oppressive atmosphere, which stifled vivacity and animation. His speech was oracular and final; and as he listened, his surcharged countenance expressed, like that of the bird of wisdom, a severe and unblenching appreciation.

"Such was, and sometimes is, the mask of ignorance; but there was much in the position of bygone generations of physicians which justified pretension. The medical man was surrounded by those who leaned upon his art with blind reliance, and exacted from him superhuman skill; and he assumed the infallibility thus spontaneously offered. Such a false position is not ours. We owe it to those who have prostrated their best energies in our cause, that there is now something to be known, for which a severe and professional demeanor is no longer a substitute. The physician may now discourse on medical subjects honestly, and hopefully; and the offspring of his knowledge, the positive amelioration of man's physical condition, justifies him in avowing the inadequacy of his art, without fear of derogating from its dignity, or of impairing confidence in himself. I well remember my surprise when a medical student, on hearing one who is eminent among us, proclaim, in answer to some medical question, that 'he did not know.' It was the ignorance of knowledge. Such downright, avowed medical ignorance, is rare; still it is worth while to learn what is to be learned, if only to avoid the error of those who, rightly believing there is much they do not know, pretend to know much that cannot be known." * * * * "The actual desire of people to be deceived has become classical; and to respond to this, in the words of Johnson, 'there is in human nature a general inclination to make people stare; and every wise man has himself to cure of it, and does cure himself.'*

"If the admiration of ignorant or weak minds is an object of your ambition, minister to this vitiated and uneducated taste; encourage credulity; invest your art with technicalities; exhibit your results, and studiously hide your methods. But if you desire to place your science upon a level with other sciences, aim with Bacon 'to make wonders plain, and not plain things wonders;'† lay it open to the world, and strip it of the repulsive garb of false pretension. Common expediency at least teaches this. The day is fast going by when the intelligent and the instructed attribute to the physician superhuman sagacity. The physician is now often asked for his premises, by people who think themselves possessed of sufficient logical abilities to follow him to his conclusions; and it is because the patients of this generation insist upon being told why, and not what, that the despotic sway of our professional ancestors is gradually escaping from our grasp. If you ever acquire in these days a reputation for infallibility, it will be after you have shown how you are infallible. All this is in accordance with the spirit of the age, which rules enlightened reason, and not ignorance, and persuades by the authority of facts, and not by its own authority.

"When, therefore, you examine a medical case, neither shake your head with the negation or the affirmation of profundity, nor indicate by your countenance the perplexity of overwhelming thought; but enter upon your duty with even an unprofessional cheerfulness, content to use the finite powers which God has given you. Spread out the facts; show which way they point. Do not say what your opinion is, but why it is so; give the evidence and all its bearings; push the diagnosis to its full extent; but remember there is a point at which the imperfection of our knowledge bars advance, and beyond which all is surmise and uncertainty. It is a common error, especially among students, to suppose that the physician must always arrive at some decision with regard to the character of a lesion or disease. It is not so, a diagnosis is often uncertain. The evidence points equally in two directions, and in such a case, ignorance alone asserts impossible knowledge."

"Content to exert a healthful influence within the immediate circle of your art, do not waste your breath in exposing error beyond the limits of real medicine; in holding up to indignation the heartlessness which supplies straws to the dying man, and tortures him with false hope, until his palsied grasp has yielded its last coin;—still less in pointing out the fallacy of those medical pseudo-sciences which last a few short years, and die of inanition; which rapidly replace each other, with nothing in common but their friends, vociferous in their

* Boswell, 1769. † Pref. to Nat. Hist.

constancy to each in health and strength, but in its waning years quietly deserting it, for some more profitable alliance. These fungous parasites of science are the natural food of weakness and credulity; a necessary to the vitiated taste of the more cultivated, or a luxury to vanity, which gratifies itself in detecting and appreciating what is obvious only to few. As well might you deprive such believers of their daily sustenance, as displace from their minds their hygienic theory. With the sound and intelligent no such attempt will be needed; with others none will avail. But in your own immediate art, scrupulously guard the barrier which professional convention has erected against the unsound in morals or in science. You cannot be too cautious in taking measures to advance your individual interest, which may be popularly confounded with those resorted to by the unprincipled.

"Introduce nostrums into every human art, but do not aspire to the notoriety which deals in secrets, and takes out letters-patent for the common offices of Christian charity. Humanity revolts at this shallow artifice of those who cannot gain the eminence ever accorded to superiority. If art is sometimes thus prostituted by those who feel no interest to maintain its dignity, thank God there is an inner temple, beyond whose precincts no sacrilegious selfishness has ever penetrated. Science radiates truth, like light, upon the world. The contemplation of the order and harmony of nature, the intercourse of truth, warms the heart, as it enlarges the intellect, and elevates man into a sphere where self is sacrificed. Great discoverers in science have been ever philanthropic; that they have shed their knowledge upon the world as freely as they have devoted their best days to its pursuit, is a fact so universal and infallible, that he who now attempts to conceal his pretended discovery, exposes to the intelligent, with his own selfishness, the fallacy of his pretensions.

"Lastly, do not lose sight of your science in your own intercourse with art. * * * * *

"Indolence, the routine of art, perhaps the demands of hard necessity, shall engross many of us in our narrow spheres, and bend us from the pursuit of knowledge. But in the struggle and turmoil of active life, do not forget your debt to science, which ever toils in quiet, unremittingly; heedless alike of its own transitory interests, and of the world around; whose highest aim is, year after year, to add its new contribution to the sum of human knowledge, which promotes by its example the best interests of our profession; which is content to find its happiness in a tranquil intercourse with the great truths of nature, and the manifestations of unerring sagacity."

J. V. Z. B.

